# Large-scale assessment of physical activity in a population using high-resolution hip-worn accelerometry: the German National Cohort (NAKO)

**DOI:** 10.1038/s41598-024-58461-5

**Published:** 2024-04-04

**Authors:** Andrea Weber, Vincent T. van Hees, Michael J. Stein, Sylvia Gastell, Karen Steindorf, Florian Herbolsheimer, Stefan Ostrzinski, Tobias Pischon, Mirko Brandes, Lilian Krist, Michael Marschollek, Karin Halina Greiser, Katharina Nimptsch, Berit Brandes, Carmen Jochem, Anja M. Sedlmeier, Klaus Berger, Hermann Brenner, Christoph Buck, Stefanie Castell, Marcus Dörr, Carina Emmel, Beate Fischer, Claudia Flexeder, Volker Harth, Antje Hebestreit, Jana-Kristin Heise, Bernd Holleczek, Thomas Keil, Lena Koch-Gallenkamp, Wolfgang Lieb, Claudia Meinke-Franze, Karin B. Michels, Rafael Mikolajczyk, Alexander Kluttig, Nadia Obi, Annette Peters, Börge Schmidt, Sabine Schipf, Matthias B. Schulze, Henning Teismann, Sabina Waniek, Stefan N. Willich, Michael F. Leitzmann, Hansjörg Baurecht

**Affiliations:** 1 Department of Epidemiology and Preventive Medicine, University of Regensburg, Franz-Josef-Strauß-Allee 11, 93053 Regensburg, Germany; 2 Accelting, Almere, The Netherlands; 3 NAKO Study Center, German Institute of Human Nutrition Potsdam-Rehbruecke, Nuthetal, Germany; 4 Division of Physical Activity, Prevention and Cancer, German Cancer Research Center (DKFZ), Im Neuenheimer Feld 581, 69120 Heidelberg, Germany; 5 Institute for Community Medicine, University Medicine Greifswald, Greifswald, Germany; 6 Molecular Epidemiology Research Group, Max-Delbrueck-Center for Molecular Medicine in the Helmholtz Association (MDC), Berlin, Germany; 7 Leibniz Institute for Prevention Research and Epidemiology – BIPS, Bremen, Germany; 8 Institute of Social Medicine, Epidemiology and Health Economics, Charité-Universitätsmedizin Berlin, Charitéplatz 1, 10098 Berlin, Germany; 9 Hannover Medical School, Peter L. Reichertz Institute for Medical Informatics, Carl-Neuberg-Strasse 1, 30625 Hannover, Germany; 10 Division of Cancer Epidemiology, German Cancer Research Center (DKFZ), Im Neuenheimer Feld 581, 69120 Heidelberg, Germany; 11 Institute of Epidemiology and Social Medicine, University of Münster, Münster, Germany; 12 Division of Clinical Epidemiology and Aging Research, German Cancer Research Center (DKFZ), Heidelberg, Germany; 13 Department for Epidemiology, Helmholtz Centre for Infection Research (HZI), Brunswick, Germany; 14 Department of Internal Medicine B, University Medicine Greifswald, Greifswald, Germany; 15 German Center for Cardiovascular Research (DZHK), Partner Site Greifswald, Greifswald, Germany; 16 Institute for Medical Informatics, Biometry and Epidemiology, University Hospital Essen, University Duisburg-Essen, Essen, Germany; 17 Institute of Epidemiology, Helmholtz Zentrum München - German Research Center for Environmental Health (GmbH), Neuherberg, Germany; 18 Institute and Clinic for Occupational, Social and Environmental Medicine, University Hospital, Ludwig-Maximilians-Universität München, Munich, Germany; 19 Institute for Occupational and Maritime Medicine Hamburg (ZfAM), University Medical Center Hamburg-Eppendorf (UKE), Seewartenstraße 10, 20459 Hamburg, Germany; 20 Saarland Cancer Registry, Saarbrücken, Germany; 21 Institute of Clinical Epidemiology and Biometry, University of Würzburg, Würzburg, Germany; 22 State Institute of Health I, Bavarian Health and Food Safety Authority, Erlangen, Germany; 23 Institute of Epidemiology, Kiel University, Kiel, Germany; 24 Institute for Prevention and Cancer Epidemiology, Faculty of Medicine and Medical Center, University of Freiburg, Freiburg, Germany; 25 Institute for Medical Epidemiology, Biometrics, and Informatics, Medical Faculty of the Martin-Luther-University Halle-Wittenberg, Halle (Saale), Germany; 26 Chair of Epidemiology, Institute for Medical Information Processing, Biometry and Epidemiology, Ludwig-Maximilians-Universität München, Munich, Germany; 27 Department of Molecular Epidemiology, German Institute of Human Nutrition Potsdam-Rehbruecke, Nuthetal, Germany; 28 Institute of Nutritional Science, University of Potsdam, Nuthetal, Germany

**Keywords:** Risk factors, Public health, Scientific data, Epidemiology

## Abstract

Large population-based cohort studies utilizing device-based measures of physical activity are crucial to close important research gaps regarding the potential protective effects of physical activity on chronic diseases. The present study details the quality control processes and the derivation of physical activity metrics from 100 Hz accelerometer data collected in the German National Cohort (NAKO). During the 2014 to 2019 baseline assessment, a subsample of NAKO participants wore a triaxial ActiGraph accelerometer on their right hip for seven consecutive days. Auto-calibration, signal feature calculations including Euclidean Norm Minus One (ENMO) and Mean Amplitude Deviation (MAD), identification of non-wear time, and imputation, were conducted using the R package GGIR version 2.10-3. A total of 73,334 participants contributed data for accelerometry analysis, of whom 63,236 provided valid data. The average ENMO was 11.7 ± 3.7 m*g* (milli gravitational acceleration) and the average MAD was 19.9 ± 6.1 m*g*. Notably, acceleration summary metrics were higher in men than women and diminished with increasing age. Work generated in the present study will facilitate harmonized analysis, reproducibility, and utilization of NAKO accelerometry data. The NAKO accelerometry dataset represents a valuable asset for physical activity research and will be accessible through a specified application process.

## Introduction

Large epidemiologic studies have established the health-promoting effects of self-reported physical activity on numerous disease endpoints, including cardiovascular disease, hypertension, type 2 diabetes, various cancers, and premature mortality^1^. However, self-report physical activity methods are fraught with numerous limitations, including susceptibility to systematic errors like social desirability reporting and recall issues, which can lead to potentially biased estimates^2,3^. By comparison, device-based measurement methods such as accelerometers provide objective estimates of physical activity^4^. Moreover, advancements in technology have made it possible to record and store triaxial raw 24-h accelerometry data over extended periods, spanning days or weeks, in large-scale studies involving thousands of participants (e.g., UK Biobank, NHANES, the HUNT4-N Study, the Pelotas Birth Cohorts)^5–8^. The major advantage of high-resolution accelerometry data lies in its capacity for transparent and flexible processing, which facilitates comparability and improves harmonization across different studies and devices^9–12^. Furthermore, data from wearable sensors enable the use of sophisticated analytical methods, such as deriving physical activity patterns and conducting compositional 24-h activity analyses^13–17^. The initial and most critical step in effectively utilizing raw accelerometry data involves rigorous quality control and the thorough generation of derived physical activity metrics.

The primary aim of the current manuscript was to describe the methodology for processing raw accelerometry data used in the German National Cohort (NAKO). Our goal was to evaluate the completeness and plausibility of the data and to provide justification for key data processing and analysis decisions. The ultimate goal was to produce and share a comprehensive repository of accelerometry data, which has the potential to address unresolved questions in the field of physical activity epidemiology.

## Methods

### Study population

Detailed information on the design and aims of the NAKO have been published elsewhere^18^. In short, 205,415 men and women (50% each) aged 19–74 years were recruited in 18 study regions in Germany at study baseline between 2014 and 2019^18^. The study aims to identify risk and protective factors as well as to provide imaging and biomarkers for major chronic diseases^18^. The NAKO is performed in accordance with the ethical standards of the institutional and/or national research committee, with national law and with the Declaration of Helsinki of 1975 (in the current, revised version). The study was approved by the responsible local ethics committees of the German Federal States where all study centers were located (Bayerische Landesärztekammer (protocol code 13023, Approval Date: 27 March 2013 and 14 February 2014 (rectification of documents, study protocol, consent form etc.))). Written informed consent was obtained from all participants^18,19^. This study is reported according to the STROBE (Strengthening the Reporting of Observational studies in Epidemiology) Guidelines/methodology (Supplementary Information 2).

### Accelerometer and data collection

ActiGraph (Pensacola, FL, USA) accelerometers have been extensively employed in epidemiologic research^20^. As a result of the evolution of ActiGraph products throughout the course of the NAKO baseline data collection, three ActiGraph models (GT3X+, wGT3X+, and wGT3X-BT) were employed. All models record acceleration (*g* (gravitational acceleration) ≈ 9.81 m/s^2^) tri-axially (in longitudinal, lateral, and anteroposterior direction when positioned on the side of the hip) and were configured at a 100 Hz sampling rate. While GT3X+ and wGT3x+ have a 6 ± *g* dynamic range, wGT3X-BT has an 8 ± *g* dynamic range (all models use a 12-bit conversion). Further, firmware versions ranged from v1.2.0 to v3.2.1. To maximize the transparency and reproducibility of data processing, we disabled the default Low-Power-Mode filter to avoid relying on manufacturer-dependent pre-processing steps of the data.

During the study center visit, study personnel attached the device above the right hip on the mid-axillary line using an elastic strap wearable over or underneath clothing. Participants were instructed to wear the device continuously (24/7) and to carry out all activities as usual. The device had to be taken off only in case of water contact lasting longer than 30 min such as in the sauna or while swimming or diving. The recording period started on the first day and ended on the eighth day after the study center visit. On the morning of day nine, the device had to be detached and sent back to the study center using a pre-paid envelope.

### Data processing

We used the open-source R package GGIR version 2.10-3^21,22^ combined with the R package read.gt3x version 1.2.0^23^ for data processing. GGIR has been described elsewhere in detail^21^. All data processing was conducted on the high-performance computing cluster at the University of Regensburg. In the following section we summarize the main processing steps.

Briefly, calibration correction coefficients were derived from non-movement periods in the acceleration data, where an iterative change point algorithm was used to calibrate signals to 1 *g*^24^. Measurements with a calibration error greater than 0.02 *g* were excluded from analysis based on our observation that the distribution of acceleration metrics values, as detailed below, showed greater variation with > 0.02 *g* calibration error compared with 0.01–0.02 *g* calibration error.

To empirically verify variation in the X and Y axis order, the longitudinal axis was estimated from the data by calculating the correlation of the epoch-by-epoch angle of each accelerometer axis with a 24-h lagged version of itself. See legend of Fig. S5 for details on angle calculation. As the longitudinal axis shows a clear upright (daytime)–lying (nighttime) pattern, it is expected to show the highest day-by-day correlation.

Non-wear time was detected using a previously described and commonly used procedure^21,25–28^. Briefly, non-wear times per 15-min interval were identified using the standard deviation (< 13.0 m*g* for ActiGraph) and the range of values (< 50.0 m*g*) of the enclosing 60-min interval^25^.

Various summary metrics were calculated per 5 s epoch, including the Euclidean norm minus one (ENMO)^25^ and the Mean Amplitude Deviation (MAD)^29^. Both metrics have previously been used in physical activity research^5,26–28,30,31^ and a detailed description can be found in Supplementary Methods S1 and S2. In contrast to some other studies^32^, no low-pass frequency filter was applied because high signal frequencies can contain harmonics of movements at lower frequencies, which is not noise but a true reflection of movement. ENMO and MAD values were aggregated separately per participant across all valid (week(end))-days, per valid day, and on a 15-min level across all valid days. Signal features were imputed for 15-min time segments classified as non-wear time or where more than 80% of the raw data points in the segment had a value close to or at the edges of the accelerometer’s dynamic range, as discussed in more detail in a previous study^25^. Note that not imputing implies zero movement and omitting the data points implies imputing using the rest of the recording.

To represent the distribution of a participant’s time spent in physical activity intensities, value distributions in 10 m*g* increments for both the ENMO and MAD metrics were derived. It must be noted that MAD values are known to be higher than ENMO values, therefore time spent in certain acceleration ranges is not directly comparable between ENMO and MAD metrics. The definition of moderate to vigorous-intensity physical activity (MVPA), i.e., physical activity conducted at an intensity of ≥ 3 metabolic equivalents of task (METs)^33^, does not lend itself to be unambiguously estimated from accelerometry data. However, MVPA is frequently used in physical activity research. Thus, to represent time spent above different physical activity intensity (acceleration) levels, various estimates were derived based on different epoch lengths, acceleration thresholds, and bout duration criteria to provide a variety of choices for data analysis and sensitivity analyses (Supplement Box 1).

In line with literature that has relied on 24-h wear protocols^34,35^, we considered days with at least 16 valid wear hours as valid. Furthermore, using 16 h (2/3 of the anticipated 24-h wear period) aligns with traditional hip worn accelerometer literature, where accelerometers were typically worn only during waking hours, with an expectation of 10 h of wear out of the approximately 15 waking hours^3^.

### Descriptive and exploratory analyses

We excluded participants with less than one valid weekend day or fewer than two valid weekdays, ensuring coverage of both weekend and weekday activities, or participants with an incomplete 24-h cycle, i.e., participants with consistently invalid data for any 15-min period across all recording days. To support that rationale, we ran missing data simulations in a subsample of 51,998 participants with perfect wear time compliance (seven valid days, five valid weekdays, two valid weekend days). Consecutively, ENMO measures from six to one random day(s) of this sample were averaged and compared against the 7-day ENMO average using intraclass correlation coefficients (ICC). The same procedure was applied to the five-weekday ENMO average and the weekend ENMO average as well as the MAD measures.

ENMO and MAD values were winsorized at the age- and sex-specific 99.9th percentile. Age was categorized according to 10-year increments. Due to a potential gap of several years between recruitment into the study and the baseline examination, the final sample contains participants older than 70 years. Biological sex was categorized as woman or man. Body height and weight were measured using a Stadiometer 274 and a medical Body Composition Analyzer 515 (seca GmbH & Co. KG, Hamburg), respectively, and BMI was classified according to WHO categories: < 18.5 kg/m^2^ as underweight, 18.5–24.9 kg/m^2^ as normal weight, 25.0–29.9 kg/m^2^ as overweight, and ≥ 30.0 kg/m^2^ as obese^36,37^. Times of day were grouped as follows: 0:00–5:59 AM, 6:00–11:59 AM, 12:00–5:59 PM and 6:00–11:59 PM. Of note, days with time shift (due to daylight saving time) were also included, as GGIR takes this into account. The first day of accelerometer wear was used to determine the month, and the season was categorized such that spring started on 1st March. To describe the distribution and characteristics of the data, we computed accelerometer wear time and average acceleration according to population subgroups defined by age, sex, and body mass index (BMI). We also explored temporal and seasonal variation in physical activity. Descriptive and exploratory statistics were calculated using R version 4.3.1^38^.

## Results

### Participant flow

**Figure 1:**
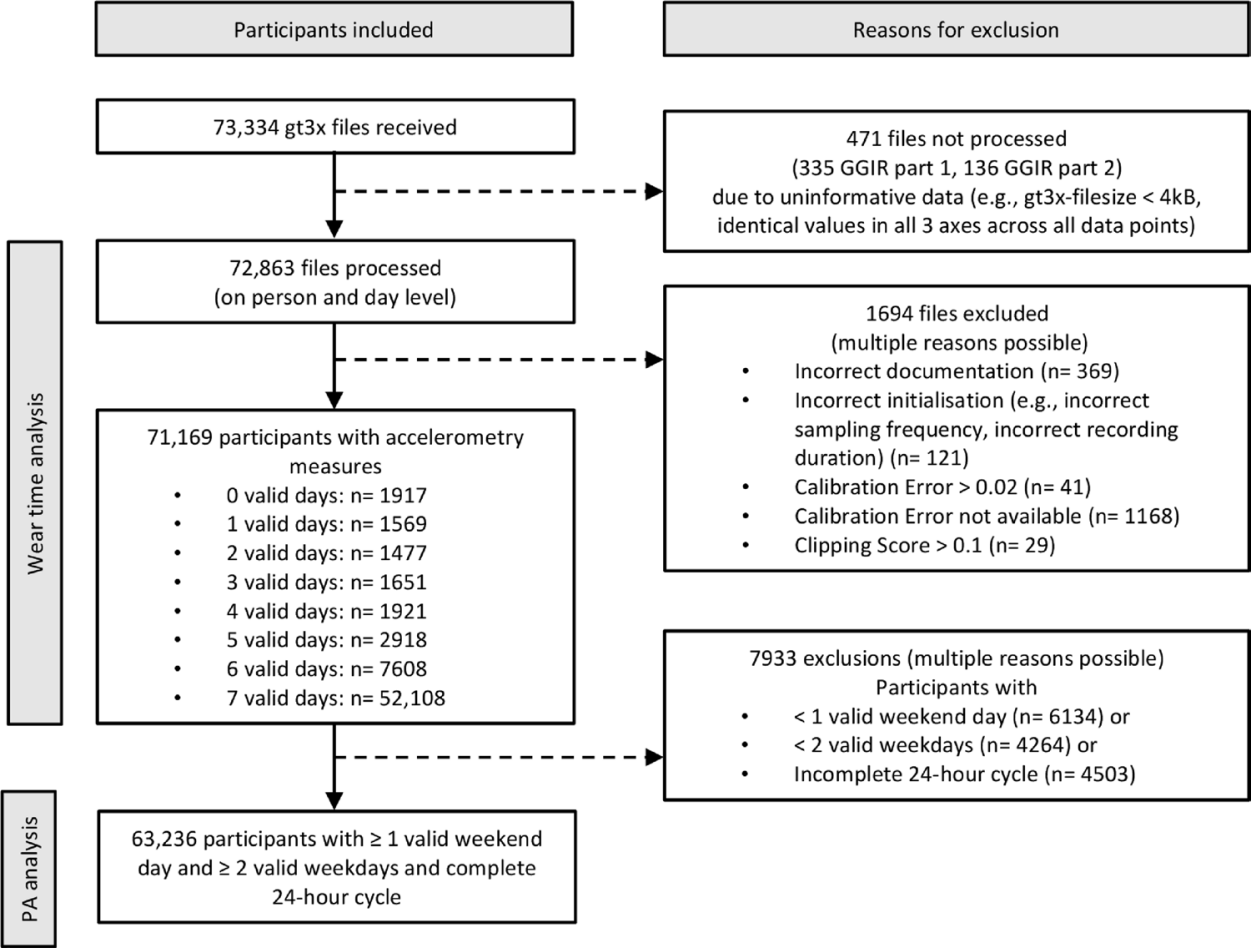
Flow chart of participants. *PA* physical activity.

We received 73,334 gt3x files, of which 471 files could not be processed due to uninformative data (e.g., file size too small). Data from further 1694 subjects were excluded due to issues that had occurred at the study centers (e.g., incorrect documentation of consent status, improper device initialization) or problems with data quality (e.g., calibration error or clipping scores exceeding threshold values). Of the resulting 71,169 individuals, we disregarded those with less than one valid weekend day, those with less than two valid weekdays, and those with an incomplete 24-h cycle, resulting in a final population for analysis of 63,236 participants (Fig. 1).

### Accelerometry wear time

Missing data simulation showed that the ICC for two valid weekdays exceeded 0.9, whether compared to the average of a 7-day period or just the 5 weekdays. Likewise, the ICC for either Saturday or Sunday surpassed 0.9 when compared to the average weekend measurement (Fig. S1). Over 90% of participants had at least four valid wear days, and compliance increased with age (Fig. S2). Median wear time was consistent across weekdays, seasons, age, BMI groups, ENMO levels, and between sexes (Table S1).

### Baseline characteristics and acceleration summary metrics

In the study, 52% of participants were women. Participants were on average 50.1 years (SD = 12.6) old and had a mean BMI of 26.4 kg/m^2^ (SD = 4.8). The average ENMO was 11.7 m*g* (SD = 3.7), with men showing slightly higher values (12.0 m*g* (SD = 4.0)) than women (11.5 m*g* (SD = 3.5)). The overall average MAD was 19.9 m*g* (SD = 6.1), with men at 20.4 m*g* (SD = 6.5) and women at 19.5 m*g* (SD = 5.7). The correlation between the winsorized (99.9th percentile) ENMO and MAD was high (r = 0.96, Supplementary Fig. S3).

**Figure 2:**
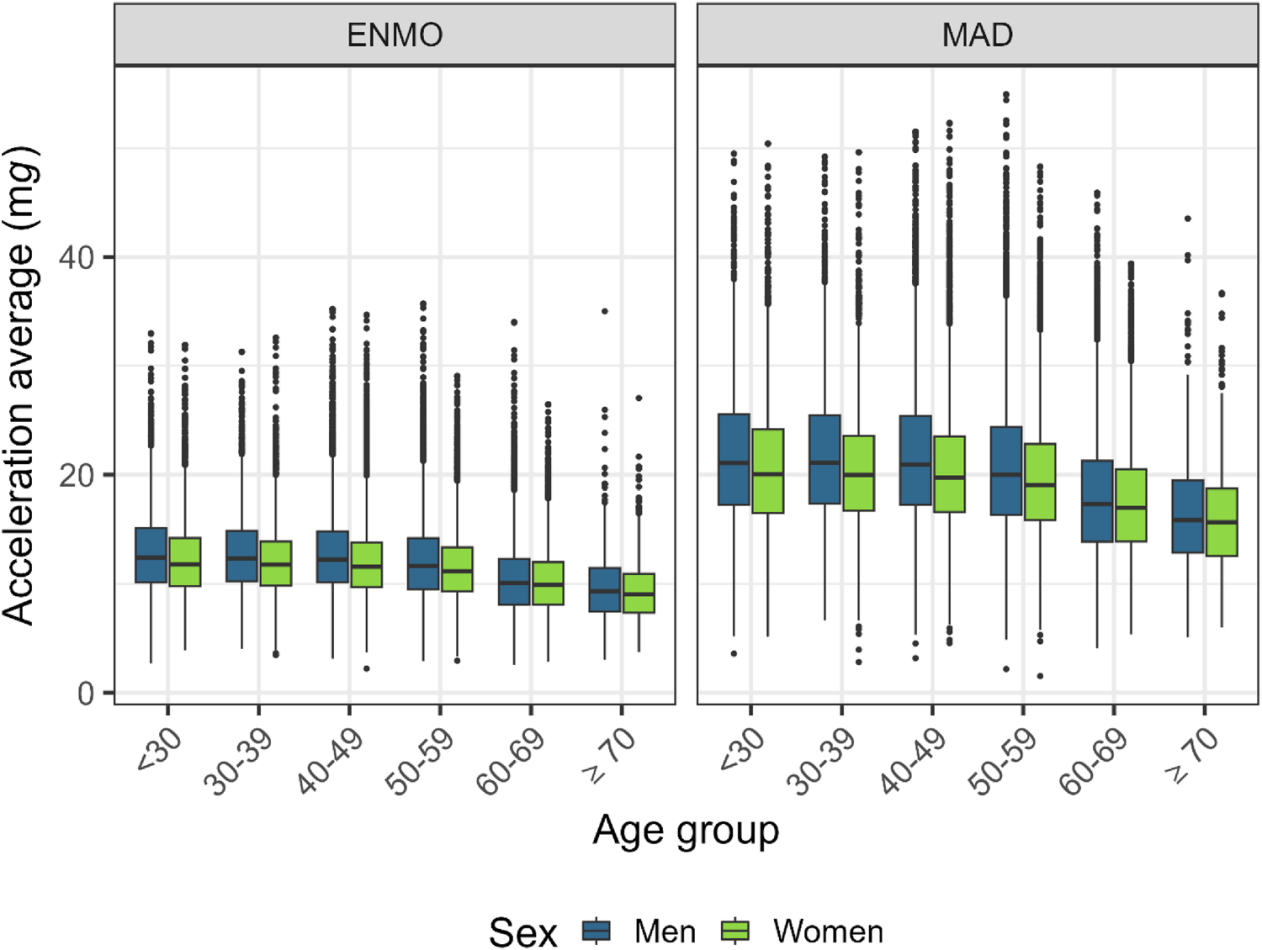
Magnitude of acceleration (ENMO and MAD) by age and sex. *ENMO* Euclidean Norm Minus One with negative values set to zero, *MAD* mean amplitude deviation, see text, *mg* milli gravitational acceleration. n = 63,236. ENMO and MAD values were winsorized at age- and sex-specific 99.9th percentile. Interpretation of box and whiskers plot: The box depicts the interquartile range (IQR, central 50% of the distribution) with the 25% quantile and the 75% quantile as lower and upper limits, respectively, as well as the median (50% quantile, middle line); the lower whisker shows the smallest observation that is greater than or equal to the 25% quantile − 1.5 × IQR; the upper whisker depicts the largest observation that is less than or equal to the 75% quantile + 1.5 × IQR; the dots indicate outliers beyond the whiskers.

For both ENMO and MAD, acceleration decreased with increasing age, it was higher in men than women, and it was higher in normal weight than underweight, overweight, or obese participants (Table 1, Fig. 2).

**Table 1:** Magnitude of acceleration (ENMO and MAD) by participant characteristics.

	ENMO [mean ± SD m*g*]		MAD [mean ± SD m*g*]	
	Men	Women	Men	Women
Age (years)				
< 30	12.9 ± 4.1	12.4 ± 3.7	21.8 ± 6.7	20.8 ± 6.1
	(n = 2660)	(n = 3024)	(n = 2660)	(n = 3024)
30–39	12.9 ± 3.8	12.2 ± 3.5	21.9 ± 6.4	20.6 ± 5.6
	(n = 3046)	(n = 3498)	(n = 3046)	(n = 3498)
40–49	12.8 ± 3.9	12.1 ± 3.5	21.8 ± 6.4	20.5 ± 5.8
	(n = 7841)	(n = 8725)	(n = 7841)	(n = 8725)
50–59	12.2 ± 4.0	11.6 ± 3.3	20.8 ± 6.6	19.7 ± 5.6
	(n = 8092)	(n = 9125)	(n = 8092)	(n = 9125)
60–69	10.5 ± 3.5	10.3 ± 3.1	18.0 ± 5.9	17.5 ± 5.1
	(n = 7923)	(n = 7990)	(n = 7923)	(n = 7990)
≥ 70	9.7 ± 3.4	9.5 ± 3.0	16.5 ± 5.5	16.2 ± 4.9
	(n = 695)	(n = 617)	(n = 695)	(n = 617)
BMI (1238 NA)				
Underweight (< 18.5 kg/m^2^)	11.4 ± 4.3	12.1 ± 3.8	19.2 ± 6.9	20.6 ± 6.2
	(n = 136)	(n = 528)	(n = 136)	(n = 528)
Normal weight (18.5–24.9 kg/m^2^)	12.9 ± 4.2	12.3 ± 3.6	22.0 ± 6.8	20.9 ± 5.8
	(n = 10,027)	(n = 16,113)	(n = 10,027)	(n = 16,113)
Overweight (25.0–29.9 kg/m^2^)	12.0 ± 3.8	11.2 ± 3.1	20.4 ± 6.3	19.0 ± 5.2
	(n = 13,452)	(n = 9578)	(n = 13,452)	(n = 9578)
Obesity (≥ 30 kg/m^2^)	10.5 ± 3.5	9.8 ± 2.9	17.9 ± 5.9	16.7 ± 4.9
	(n = 6049)	(n = 6115)	(n = 6049)	(n = 6115)
Time of day^a^				
0:00–5:59 AM	3.3 ± 3.0	2.8 ± 2.4	4.9 ± 4.1	4.1 ± 2.9
	(n = 30,257)	(n = 32,979)	(n = 30,257)	(n = 32,979)
6:00–11:59 AM	14.2 ± 7.0	13.9 ± 6.2	24.4 ± 11.5	23.7 ± 10.1
	(n = 30,257)	(n = 32,979)	(n = 30,257)	(n = 32,979)
12:00–5:59 PM	18.5 ± 7.3	18.1 ± 6.4	32.2 ± 12.1	31.5 ± 10.8
	(n = 30,257)	(n = 32,979)	(n = 30,257)	(n = 32,979)
6:00–11:59 PM	11.7 ± 6.2	10.9 ± 5.2	19.7 ± 10.0	18.4 ± 8.5
	(n = 30,257)	(n = 32,979)	(n = 30,257)	(n = 32,979)
Day of the week^b^				
Week	12.1 ± 4.1	11.6 ± 3.5	20.7 ± 6.9	19.8 ± 5.9
	(n = 30,257)	(n = 32,979)	(n = 30,257)	(n = 32,979)
Weekend	11.5 ± 5.1	11.1 ± 4.4	19.6 ± 8.3	18.9 ± 7.3
	(n = 30,257)	(n = 32,979)	(n = 30,257)	(n = 32,979)
Season of the year^c^				
Spring	12.2 ± 4.0	11.6 ± 3.5	20.9 ± 6.7	19.8 ± 5.8
	(n = 7926)	(n = 8692)	(n = 7926)	(n = 8692)
Summer	12.5 ± 4.1	12.0 ± 3.6	21.3 ± 6.7	20.4 ± 5.9
	(n = 6971)	(n = 7887)	(n = 6971)	(n = 7887)
Autumn	11.8 ± 3.9	11.4 ± 3.4	20.1 ± 6.4	19.3 ± 5.6
	(n = 7558)	(n = 8483)	(n = 7558)	(n = 8483)
Winter	11.4 ± 3.8	10.9 ± 3.3	19.3 ± 6.3	18.5 ± 5.5
	(n = 7802)	(n = 7917)	(n = 7802)	(n = 7917)

n = 63,236 participants (sample for physical activity analysis).

ENMO and MAD values were winsorized at age- and sex-specific 99.9th percentile.

*BMI* body mass index, *ENMO* Euclidean Norm Minus One with negative values set to zero, *MAD* mean amplitude deviation, see text, *mg* milli gravitational acceleration, *SD* standard deviation.

^a^Average acceleration vector magnitude per time period.

^b^Average acceleration vector magnitude per day; Only valid days with ≥ 16 valid wear hours included.

^c^Spring starting on 1st March; First day of wear determines classification to month.

**Figure 3:**
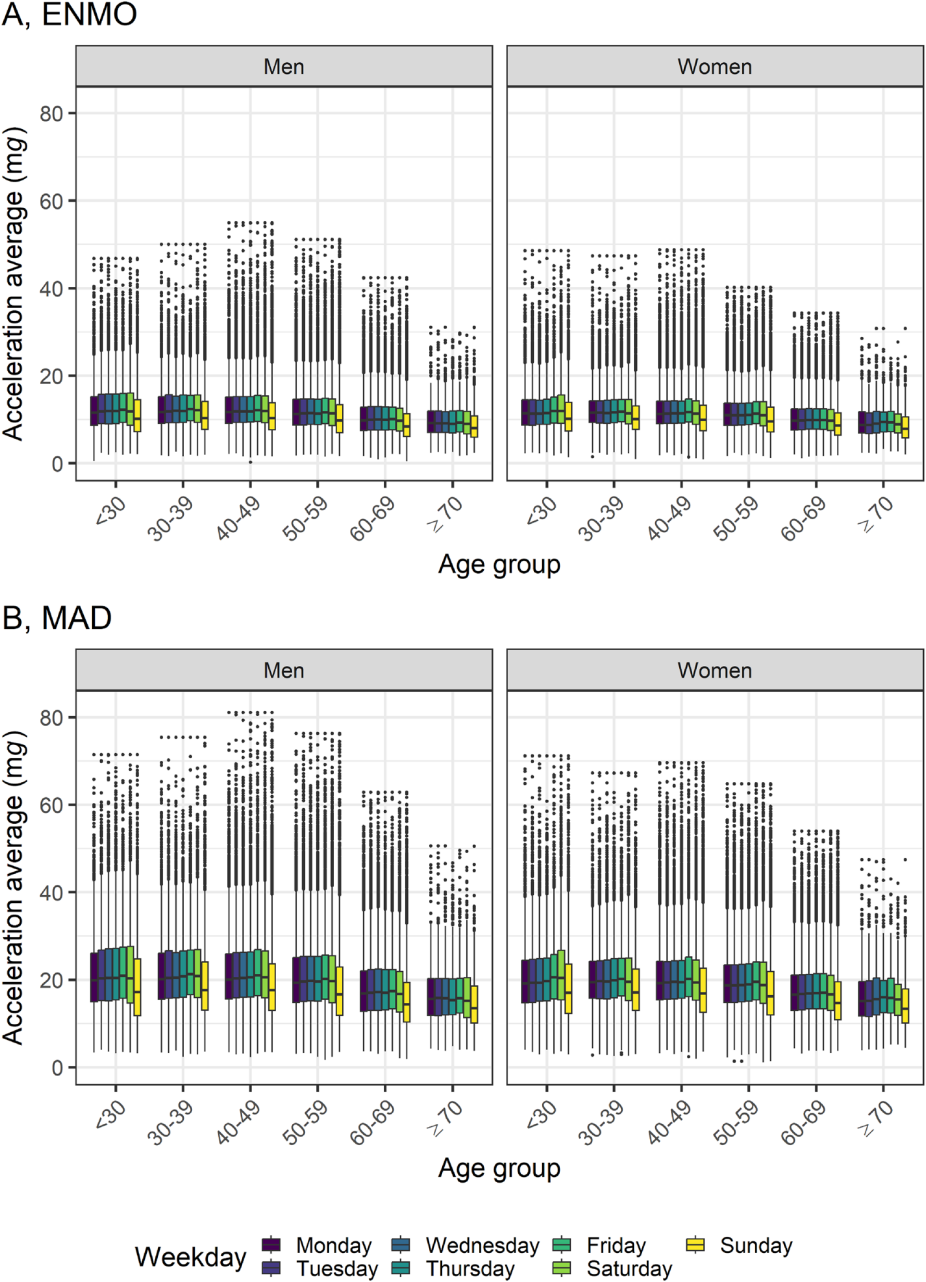
Weekday variation in magnitude of acceleration (**A**, ENMO and **B**, MAD) by age, and sex. *ENMO* Euclidean Norm Minus One with negative values set to zero, *MAD* mean amplitude deviation, see text, *mg* milli gravitational acceleration. n = 63,236. ENMO and MAD values were winsorized at age- and sex-specific 99.9th percentile. Interpretation of box and whiskers plot: The box depicts the interquartile range (IQR, central 50% of the distribution) with the 25% quantile and the 75% quantile as lower and upper limits, respectively, as well as the median (50% quantile, middle line); the lower whisker shows the smallest observation that is greater than or equal to the 25% quantile − 1.5 × IQR; the upper whisker depicts the largest observation that is less than or equal to the 75% quantile + 1.5 × IQR; the dots indicate outliers beyond the whiskers.

**Figure 4:**
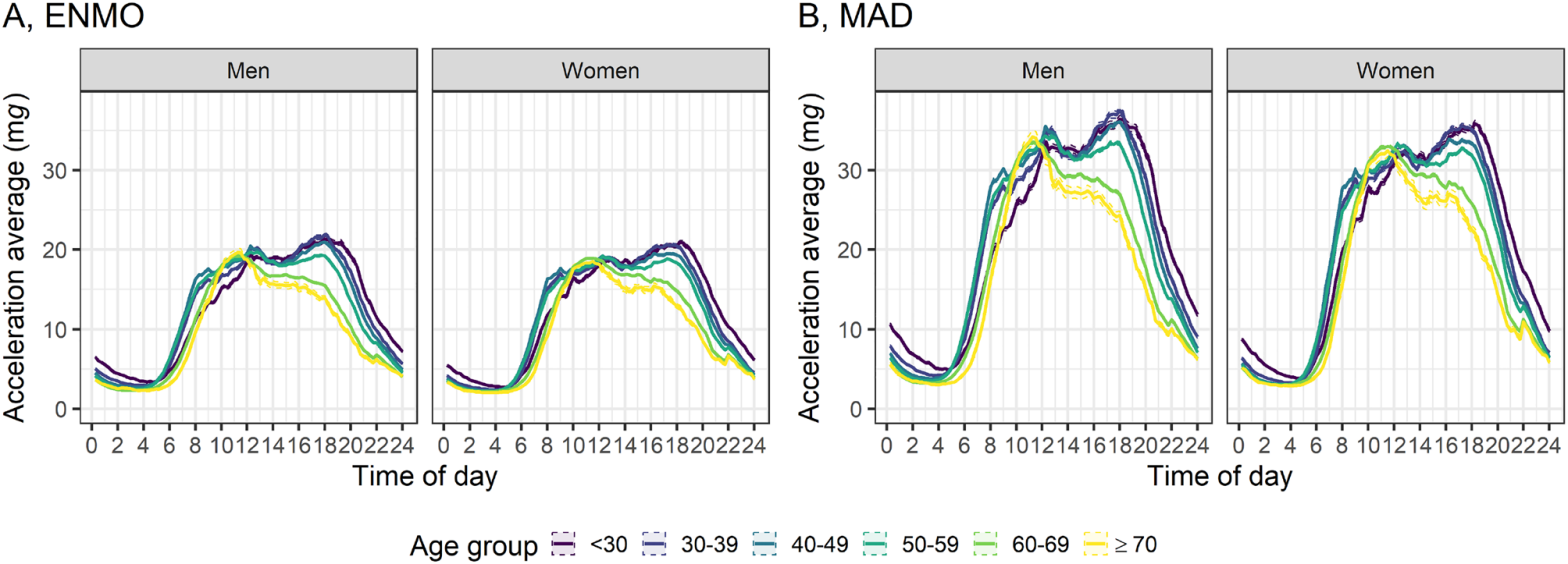
Daytime variations in magnitude of acceleration (**A**, ENMO and **B**, MAD) by age and sex. *ENMO* Euclidean Norm Minus One with negative values set to zero, *MAD* mean amplitude deviation, see text, *mg* milli gravitational acceleration. n = 63,236. ENMO and MAD values were winsorized at age- and sex-specific 99.9th percentile. Shading bounds represent two standard errors.

Acceleration levels were higher on weekdays compared to weekend days (Table 1). Notably, physical activity levels were distinctly lower on Sundays for both men and women across all age groups (Fig. 3). Additionally, both ENMO and MAD values peaked in the summer and were lowest in the winter, affecting both men and women in most age groups (Table 1, Fig. S4). ENMO and MAD values were highest between 12:00 PM and 05:59 PM, and lowest between 0:00 AM and 5:59 AM (Table 1). Younger participants were physically more active in the evening, whereas older subjects were physically more active in the morning (Fig. 4). The variation in the angle of the accelerometer’s longitudinal axis, indicative of its orientation in three-dimensional space, showed a clear difference between upright posture during daytime hours and a reclined posture during nighttime (Fig. S5).

### Activity intensity categories

**Figure 5:**
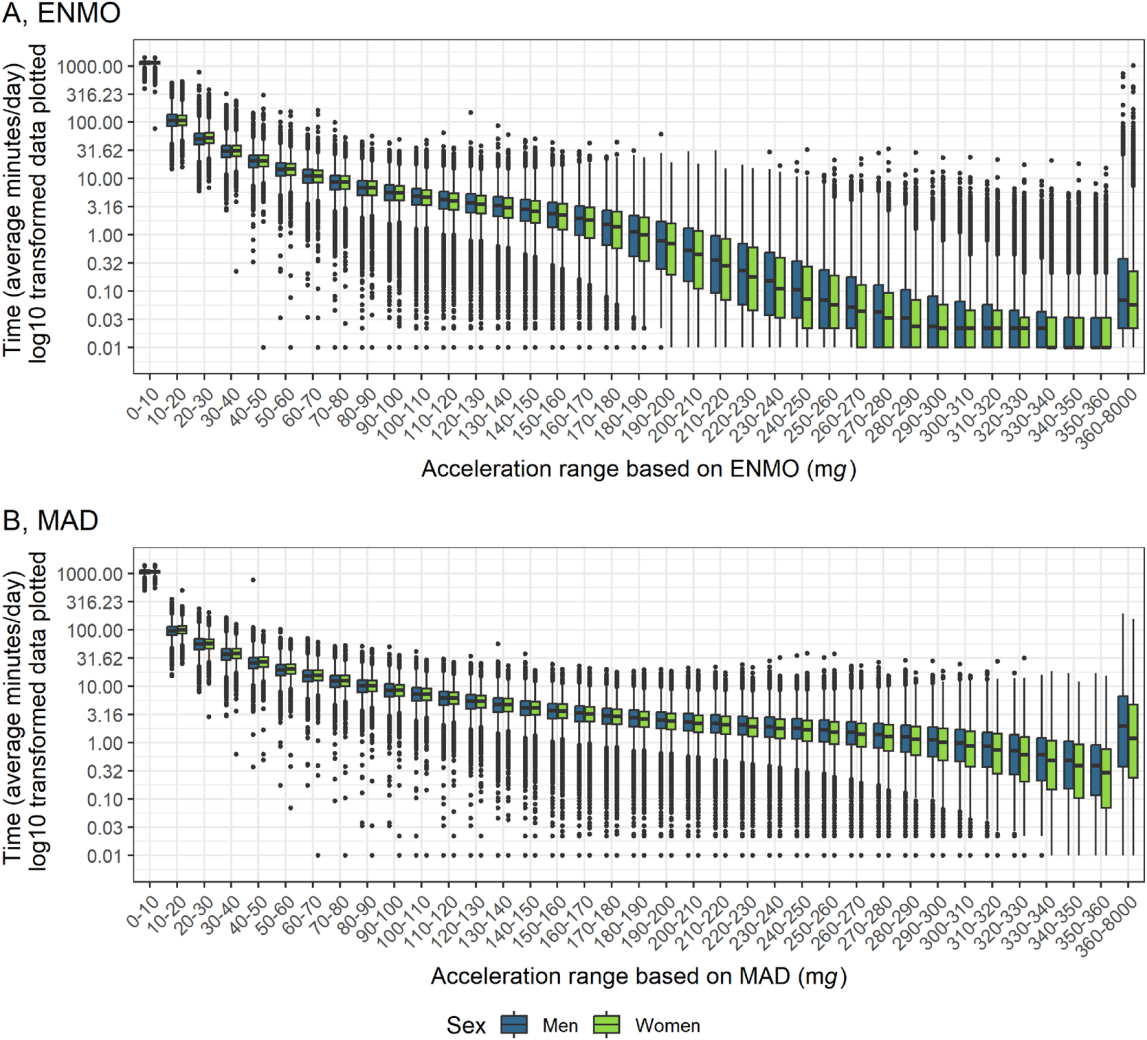
Time spent in acceleration ranges based on (**A**) ENMO and (**B**) MAD by sex. *ENMO* Euclidean Norm Minus One with negative values set to zero, *MAD* mean amplitude deviation, *mg* milli gravitational acceleration. n = 63,236. Values were not winsorized. Interpretation of box and whiskers plot: The box depicts the interquartile range (IQR, central 50% of the distribution) with the 25% quantile and the 75% quantile as lower and upper limits, respectively, as well as the median (50% quantile, middle line); the lower whisker shows the smallest observation that is greater than or equal to the 25% quantile − 1.5 × IQR; the upper whisker depicts the largest observation that is less than or equal to the 75% quantile + 1.5 × IQR; the dots indicate outliers beyond the whiskers.

Participants predominantly spent their time in the lowest activity intensity category, ranging from 0 to 10 m*g* of ENMO or MAD (Fig. 5A,B). Time spent in any given acceleration category decreased with increasing activity intensity.

**Figure 6:**
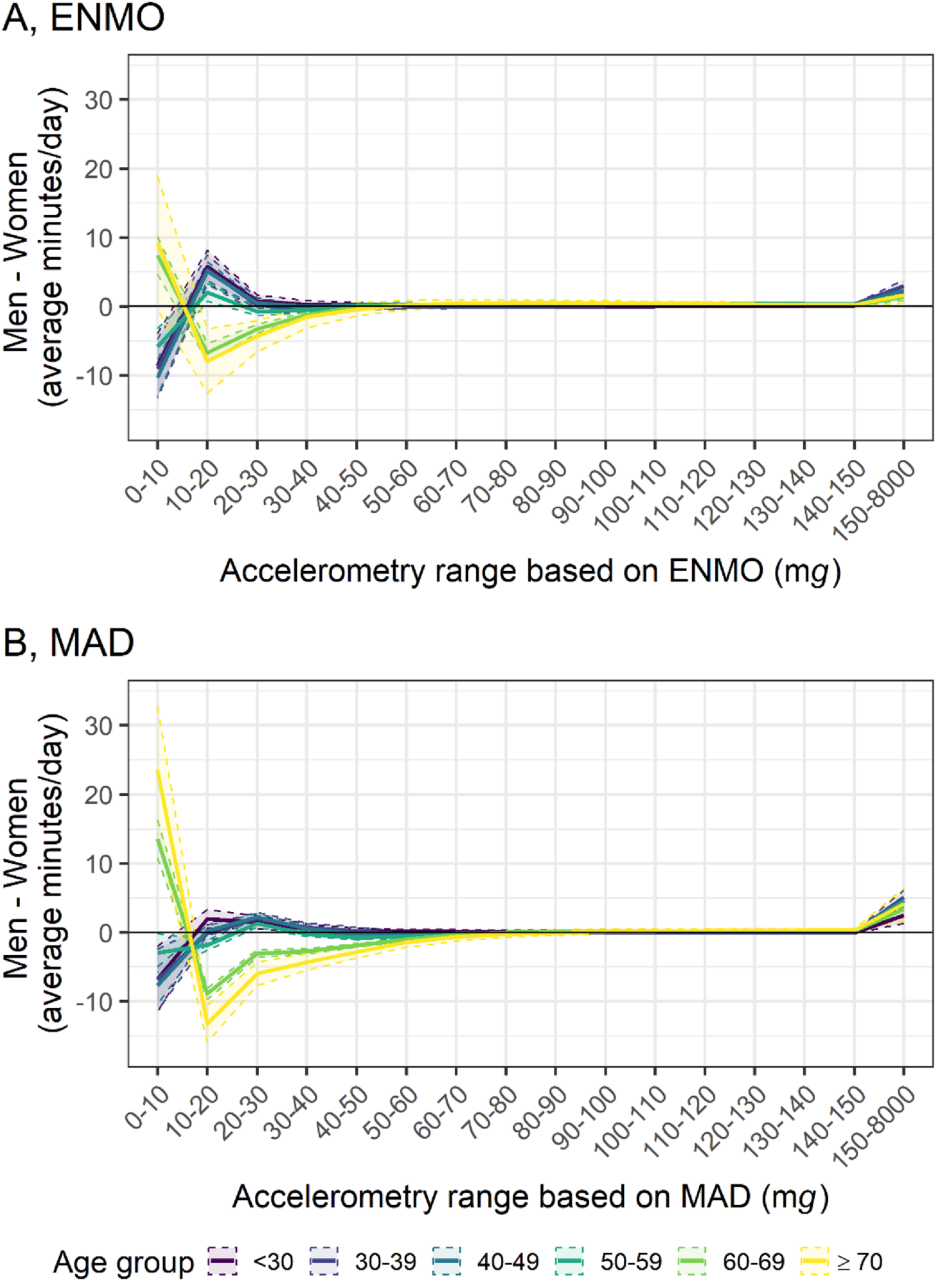
Sex-differences of time spent in acceleration ranges based on (**A**) ENMO and (**B**) MAD by age. *ENMO* Euclidean Norm Minus One with negative values set to zero, *MAD* mean amplitude deviation, *mg* milli gravitational acceleration. n = 63,236. Lines represent the difference of the mean time (minutes per day) spent in acceleration categories in the group of men and the group of women. Shading bounds represent the 95% confidence interval of the two-sample t-test with the Null hypothesis: “true difference in means between group men and group women is equal to zero”. Values were not winsorized.

ENMO-based analyses showed that women below age 60 years spent more time in the lowest intensity category (0–10 m*g*) and less time in the 10–20 m*g* intensity category than men. Conversely, women over age 60 years spent less time in the 0–10 m*g* category and more time in the 10–30 m*g* categories than men (Fig. 6A). MAD-based analyses showed a similar pattern, with the differences being more pronounced among participants over age 60 years and less so in those under age 60 (Fig. 6B).

Time spent above various activity intensity thresholds, calculated using distinct algorithms (Box S1) is plotted in Fig. S6. Using a 1-min epoch without bout detection and applying age-specific cut-points from literature, participants under 60 years averaged 45.5 min per day (SD = 27.2) in MVPA based on the 70 m*g* ENMO threshold^39^, and 72.7 min per day (SD = 34.8) based on the 90 m*g* MAD threshold^40^.

## Discussion

The NAKO collected raw, seven-day, hip-worn accelerometry data, providing plausible estimates of physical activity from over 63,000 highly adherent participants. Our derived accelerometry summary metrics showed higher physical activity in men than women, declining activity with increasing age, and temporal variation reflecting rest-activity rhythms as well as higher physical activity on working days (Monday to Saturday) versus Sundays. The derived variables are available in four levels of detail, each catering to different research needs**:** first, individual-level data aggregated across all valid days for overall physical activity analyses; second, week segment-level data for comparing physical activity between weekdays and weekend days; third, day-level data, aggregated by valid day, for comparing days of the week; and fourth, 15-min-level data, aggregated across all valid days, for detailed temporal analyses of physical activity.

Comparing our data to the literature poses challenges because accelerometry study protocols vary due to different practical requirements. For example, in some studies accelerometers were only worn during waking hours. Other large epidemiologic studies such as NHANES (2011–2014), the UK Biobank, the Whitehall II Study, or the Pelotas Birth Cohorts^5,6,8,34^ used wrist worn accelerometers, as this promises superior wear compliance^41,42^ and is more broadly accepted in sleep research^43^. Since the wrist is exposed to stronger accelerations than the hip, wrist worn accelerometers yield higher activity values than accelerometers worn at the hip^35^. For example, in the UK Biobank, age- and sex-specific ENMO values measured at the wrist ranged between 22.9 and 31.2 m*g*^5^, while in our study, ENMO values measured at the hip ranged between 9.5 and 12.9 m*g*. Recent studies utilizing ActiGraph GT3X+ devices worn at the hip align with our results. In a secondary analysis of data from 220 participants of the Iowa Bone Development Study (IBDS), the mean ENMO value was 15.5 m*g* (SD = 3.9)^44^. A Spanish study involving 209 men and women found an average ENMO of 11.5 m*g* (SD = 3.2)^45^. Another Spanish study with 42 participants reported an average ENMO of 16.0 m*g* (SD = 5.6) and an average MAD of 24.4 m*g* (SD = 6.9)^35^.

We excluded 7933 participants (11%) who had less than one valid weekend day, less than two valid weekdays, or an incomplete 24-h cycle to capture both weekend and weekday behavior. This exclusion rate aligns with other large-scale studies, like the UK Biobank, which also excluded 7% of data due to insufficient wear time^5^.

Auto-calibration was originally designed for wrist-worn accelerometry data and is less suitable for hip-worn accelerometry due to the reduced sensor orientation variability at the hip. Therefore, caution is advised when analyzing data from individuals with high acceleration levels or extended periods of non-wear time. Despite our stringent exclusion criteria, our dataset still included outliers with extreme values for time spent in MVPA or average acceleration, possibly due to calibration issues or sensor malfunctions. To address this, we recommend winsorizing such outliers at the 99.9th percentile.

We used raw accelerometry data to derive ENMO and MAD, two body acceleration summary metrics that possess certain assumptions. ENMO assumes well-calibrated sensor data with continuous representation of gravitational acceleration as 1 *g*. MAD assumes that the epoch mean of the signal vector reflects the gravitational acceleration and that its oscillations are always below 0.2 Hz (1 divided by MAD window length of 5 s). Nonetheless, both metrics deliver information on movement kinematics. ENMO is correlated with energy expenditure and has associations with demographic variables and health outcomes in studies using wrist-worn accelerometers like the UK Biobank and Pelotas Birth Cohorts^5,8,25^. In our dataset, ENMO and MAD measures were highly correlated and showed similar patterns across age and sex groups. However, MAD may represent a superior metric for hip-worn accelerometer data due to its lower sensitivity to calibration errors^24,35^. Of note, MAD values are known to be higher than ENMO values, thus both metrics are in certain acceleration ranges not directly comparable. Also, divergent error structures and different sensitivities to true body movement exist between ENMO and MAD. For example, ENMO may show larger error for sedentary behavior when the accelerometer is not well calibrated. Similarly, MAD may overestimate acceleration when the accelerometer rotates with frequencies that have a time period shorter than the epoch length. We focused on ENMO and MAD because those two metrics are the most extensively researched, they are easy to document, are computationally fast, have values expressed in units of gravity, and are sufficient for descriptive quality assessment as is the focus of our current investigation. We acknowledge that other metrics may prove equally valuable, and this should be explored in future studies using NAKO data as the number of possible metrics is large.

MVPA lacks a clear, measurable definition, and acceleration does not necessarily correlate with MET-based activity intensities. Also, cut points used to classify time spent in sedentary behavior, light physical activity, and MVPA have typically been derived using small study samples, making them less transferable to all populations or age groups. Additionally, the concept of MVPA varies depending on whether cut points are based on wrist or hip data^46^. Furthermore, categorizing continuous data leads to information loss, reduced precision, and potential bias^47^. Therefore, the focus in public health should be on encouraging overall physical activity rather than maximizing time spent above certain thresholds^48^. However, the concept of measuring time spent in MVPA is well established and widely used in accelerometry data analysis. To offer flexibility for future studies, we derived a range of MVPA estimates using literature-based thresholds^39,40,49–51^. For example, researchers could consider assessing time spent in MVPA using 1-min epochs without bout detection, employing literature-based thresholds and surrounding values as a sensitivity analysis. Although, in the age group over 70 some thresholds for ENMO as proposed in the literature are concerningly close to zero^51,52^ complicating a reliable distinction between variations caused by calibration error and variations caused by time in MVPA. In this case, we recommend either using higher thresholds or focussing on the full distribution of MAD metric values.

Our study has several important strengths. We produced an elaborate set of physical activity metrics using open-source software with transparent documentation of all coding steps (Table S2), which facilitates data interpretation and eases comparability with other studies. Our summary variables have been integrated into the NAKO database and are available for researchers to apply for analytical use.

However, our study has certain limitations. Wearing an accelerometer might induce reactivity effects, leading to increased physical activity, despite guidance for participants to continue their normal routines. Also, the cohort may not fully represent the general population in Germany, limiting the generalizability of our results^18^. While there are no clear recommendations for deriving MVPA measures, we have made all the necessary data available for comprehensive sensitivity analysis.

## Conclusion

The NAKO generated plausible estimates of physical activity from hip-worn accelerometry involving over 63,000 highly compliant participants. We derived a comprehensive set of summary metrics for accelerometry, enhancing the reproducibility, utilization, and interpretation of physical activity data. These variables and the raw data are valuable for future analyses exploring associations between physical activity and disease outcomes. They can be used to statistically adjust for physical activity in multivariate models, support methodological research, and potentially identify high-risk, physically inactive population subgroups. This aligns with efforts to inform intervention strategies and guide policies targeting the WHO’s Global Action Plan goal of reducing physical inactivity by 15% by 2030^53^.

### Supplementary Information

Supplementary Information 1.

Supplementary Information 2.

## Data Availability

Access to and use of NAKO data and biosamples can be obtained via an electronic application portal (https://transfer.nako.de).

## Code availability

The Code used for processing NAKO hip worn accelerometer data on the computing cluster is openly available from the Github repository at https://github.com/UREpiPrevMed/nako-accelerometry.
