# Supplementary material for: Large-scale assessment of physical activity in a population using high-resolution hip-worn accelerometry: the German National Cohort (NAKO)
Source: Sci Rep. 2024 Apr 4;14:7927. doi: 10.1038/s41598-024-58461-5 (PMC10995156; doi:10.1038/s41598-024-58461-5)
Supplement: Supplementary file 1 — Supplementary Information 1. [file 41598_2024_58461_MOESM1_ESM.pdf]

Additional File 1

Article title: Large-Scale Assessment of Physical Activity in a Population Using High-Resolution Hip-Worn Accelerometry: The German National Cohort (NAKO)

Journal name: Scientific Reports

Author names: Andrea Weber, Vincent T. van Hees, Michael J. Stein, Sylvia Gastell, Karen Steindorf, Florian Herbolsheimer, Stefan Ostrzinski, Tobias Pischon, Mirko Brandes, Lilian Krist, Michael Marschollek, Karin Halina Greiser, Katharina Nimptsch, Berit Brandes, Carmen Jochem, Anja M. Sedlmeier, Klaus Berger, Hermann Brenner, Christoph Buck, Stefanie Castell, Marcus Dörr, Carina Emmel, Beate Fischer, Claudia Flexeder, Volker Harth, Antje Hebestreit, Jana-Kristin Heise, Bernd Holleczek, Thomas Keil, Lena Koch-Gallenkamp, Wolfgang Lieb, Claudia Meinke-Franze, Karin B. Michels, Rafael Mikolajczyk, Alexander Kluttig, Nadia Obi, Annette Peters, Börge Schmidt, Sabine Schipf, Matthias B. Schulze, Henning Teismann, Sabina Waniek, Stefan N. Willich, Michael F. Leitzmann[#], Hansjörg Baurecht[#]

Corresponding author: Andrea Weber. Department of Epidemiology and Preventive Medicine, University of Regensburg, Franz-Josef-Strauß-Allee 11, 93053 Regensburg, Germany. Andrea.Weber@klinik.uni-regensburg.de

# Joint senior authors

Supplementary Methods

Supplementary Method S1 The Euclidean norm minus one (ENMO)
Supplementary Method S2 The Mean Amplitude Deviation (MAD)

Tables

Table S1 Wear time by age, BMI, day of the week, and season of the year, stratified by sex
Table S2 Checklists for transparent documentation of raw accelerometry quality control

Figures

Figure S1 Minimum wear time criterion for whole week, weekdays, and weekend days
Figure S2 Cumulative distribution function of accelerometer wear day compliance (0 – 7 days)
Figure S3 Correlation between winsorized ENMO and MAD values by age, and sex
Figure S4 Seasonal variation in magnitude of acceleration A, ENMO and B, MAD by age, and sex
Figure S5 Accelerometer orientation: variation in angle of long axis across the day by age and sex
Figure S6 Comparison of time spent in MVPA estimated using different parameters and thresholds

Box

Box S1 Parameters and thresholds used to estimate time spent in moderate to vigorous-intensity physical activity

Supplementary Method S1 The Euclidean norm minus one (ENMO)

The Euclidean norm minus one (ENMO) is a summary metric of physical activity derived from accelerometer raw data, as described elsewhere.[1] The ENMO of a certain time point is defined as the Euclidean norm for the three-dimensional acceleration at this time point with one gravitational unit being subtracted and negative values truncated to zero:

$$\mathrm{ENMO} = \max\{(\mathrm{EN} \ - 1g), \quad 0\}, \text{ with}$$
$$\mathrm{EN} = \sqrt{acc_x^2 + acc_y^2 + acc_z^2}$$

In the NAKO, ENMO values were collapsed to five-second epochs measured in milli gravity (m*g*) units. Hence, for each participant, up to approximately 120,000 ENMO values could be measured over the 7-day period.

Supplementary Method S2 The Mean Amplitude Deviation (MAD)

The Mean Amplitude Deviation (MAD) is calculated as the average of the absolute deviation of the Euclidean norm (EN) from the epoch average, as described elsewhere:[2]

$$\mathrm{MAD} = \frac{1}{n}\sum_{i=1}^{n}|\mathrm{EN}_i - \overline{\mathrm{EN}}|$$

In the NAKO, MAD values are also calculated for five-second epochs measured in milli gravity (m*g*) units.

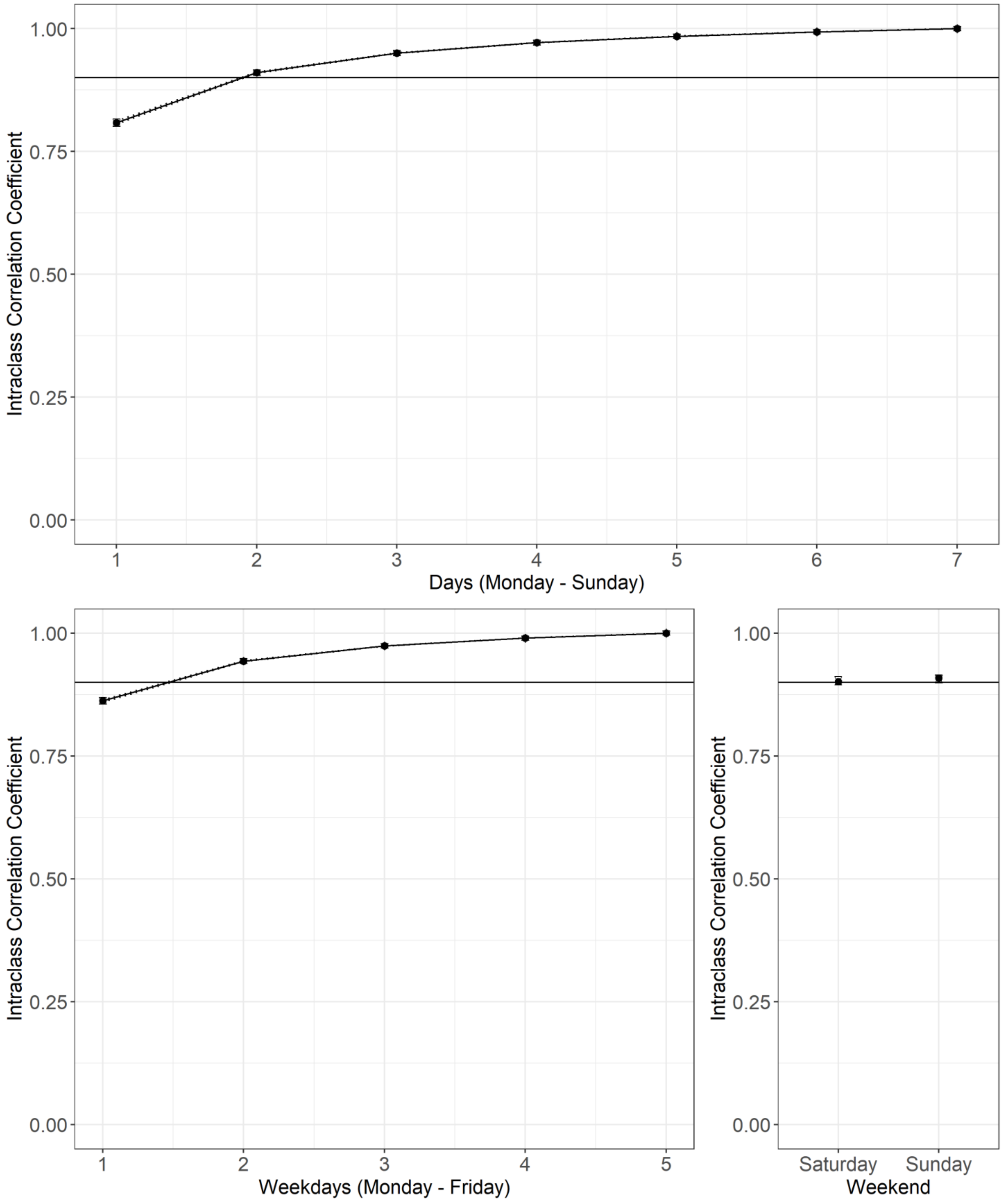

Figure S1 Minimum wear time criterion for whole week, weekdays, and weekend days

To motivate and justify our decisions, we ran missing data simulations in a subsample of 51,998 participants who had perfect wear time compliance (seven valid days, five valid weekdays, two valid weekend days). Intraclass correlation coefficients were used to determine the number of wear days needed to be within 10% of a complete seven-day measure, i.e., yielding ICC values ≥ 0.9.

Results were virtually identical for ENMO and MAD values.

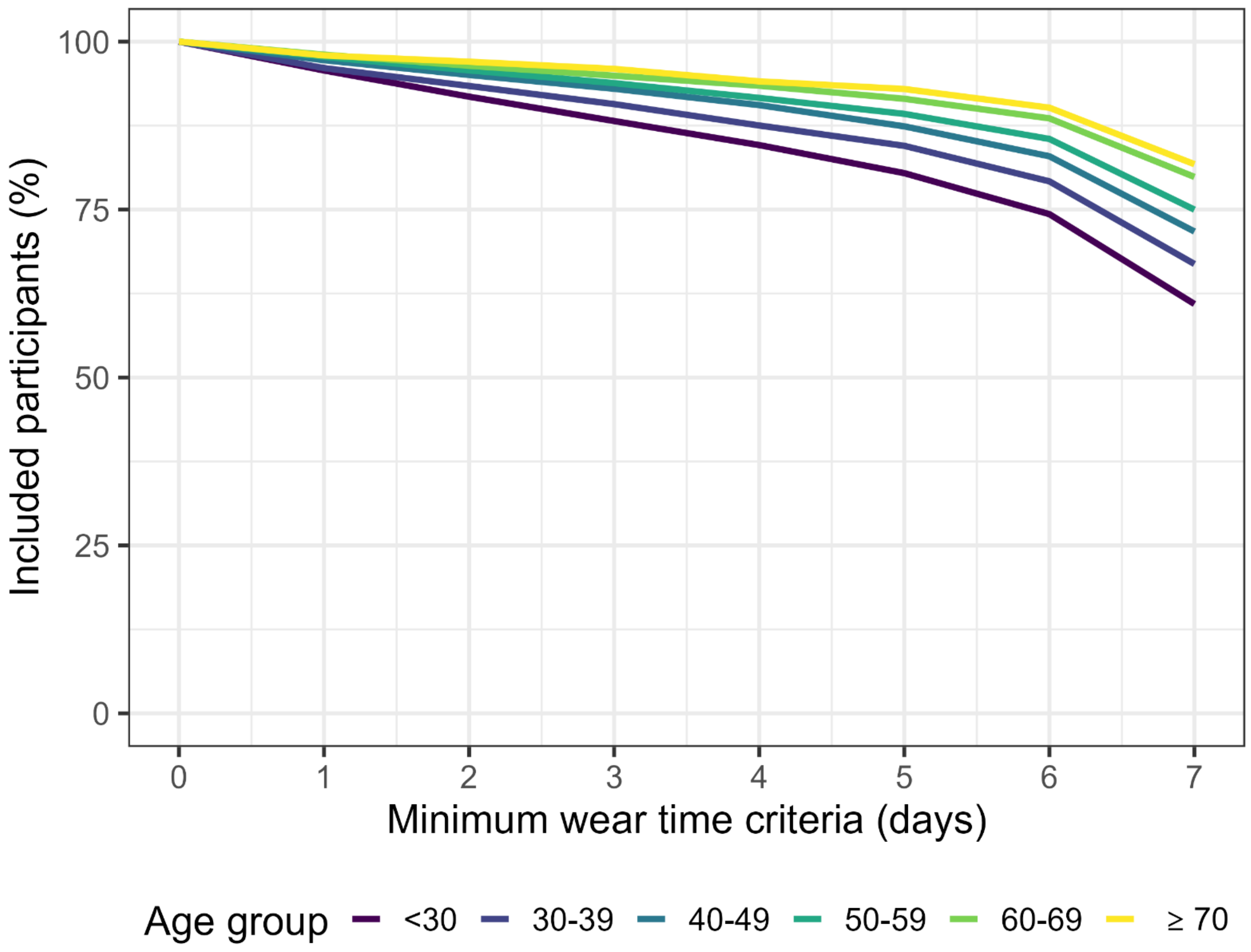

Figure S2 Cumulative distribution function of accelerometer wear day compliance (0 – 7 days)

n=71,169 participants (sample for wear time analysis)

Table S1 Wear time by age, BMI, day of the week, season of the year, and ENMO stratified by sex

	Wear time [median (IQR) hours]	
	**Men**	**Women**
**Age (years)**		
<30	162.2 (133.5-167.2) (n=3243)	164.0 (139.6-167.5) (n=3626)
30-39	163.8 (143.0-167.5) (n=3610)	165.5 (149.8-167.5) (n=4054)
40-49	165.2 (149.8-167.5) (n=8955)	165.8 (154.5-167.5) (n=9812)
50-59	166.0 (154.5-167.5) (n=9028)	166.0 (157.0-167.5) (n=10,166)
60-69	166.5 (160.2-167.5) (n=8535)	166.2 (159.5-167.5) (n=8737)
≥70	166.5 (160.5-167.5) (n=746)	166.0 (160.5-167.2) (n=657)
**BMI (1395 NA)**		
Underweight (<18.5 kg/m^2)	164.8 (150.9-167.5) (n=154)	166.5 (152.0-167.5) (n=612)
Normal weight (18.5–24.9 kg/m^2)	166.0 (153.0-167.5) (n=11,345)	166.0 (155.2-167.5) (n=18,087)
Overweight (25.0–29.9 kg/m^2)	165.8 (153.2-167.5) (n=15,118)	166.0 (156.8-167.2) (n=10,648)
Obesity (≥30 kg/m^2)	165.8 (152.2-167.5) (n=6841)	165.5 (153.5-167.2) (n=6969)
**Day of the week**[a]		
Week	23.8 (22.0-24.0) (n=34,117)	23.8 (22.2-23.9) (n=37,052)
Weekend	23.9 (23.0-24.0) (n=34,117)	23.9 (23.2-24.0) (n=37,052)
**Season of the year**[b]		
Spring	165.8 (153.2-167.5) (n=8904)	166.0 (156.2-167.2) (n=9660)
Summer	165.5 (151.5-167.5) (n=7898)	165.8 (153.0-167.5) (n=8976)
Autumn	165.8 (153.0-167.5) (n=8514)	165.8 (155.2-167.5) (n=9541)
Winter	166.0 (153.8-167.5) (n=8801)	166.0 (156.5-167.5) (n=8875)
**ENMO (1917 NA)**		
High	165.8 (153.5-167.5) (n=17,535)	166.2 (157.0-167.5) (n=17,091)
Low	166.0 (156.5-167.5) (n=15,711)	166.0 (157.5-167.5) (n=18,915)

BMI = Body Mass Index; ENMO = Euclidean Norm Minus One with negative values set to zero; IQR = interquartile range
n=71,169 participants (sample for wear time analysis)
Sum of valid wear hours per week (max = 168.0)
[a] Average valid wear time hours for day displayed (max = 25.0)
[b] Spring starting on 1st March; First day of wear determines classification to month

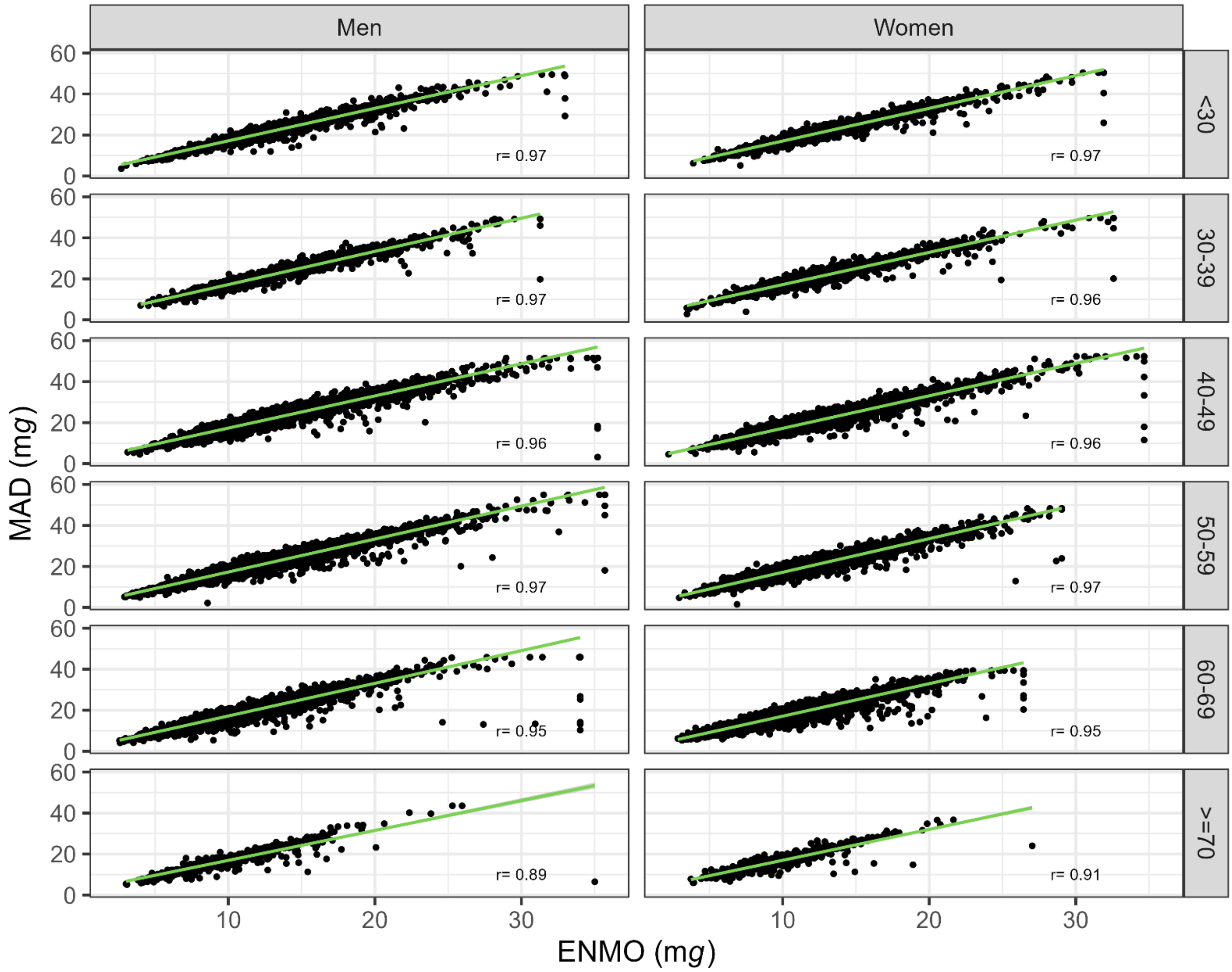

Figure S3 Correlation between winsorized ENMO and MAD values by age, and sex

ENMO = Euclidean Norm Minus One with negative values set to zero; MAD = Mean Amplitude Deviation; m*g* = milli gravitational acceleration; r = Pearson correlation coefficient

n=63,236

ENMO and MAD values were winsorized at age- and sex-specific 99.9th percentile

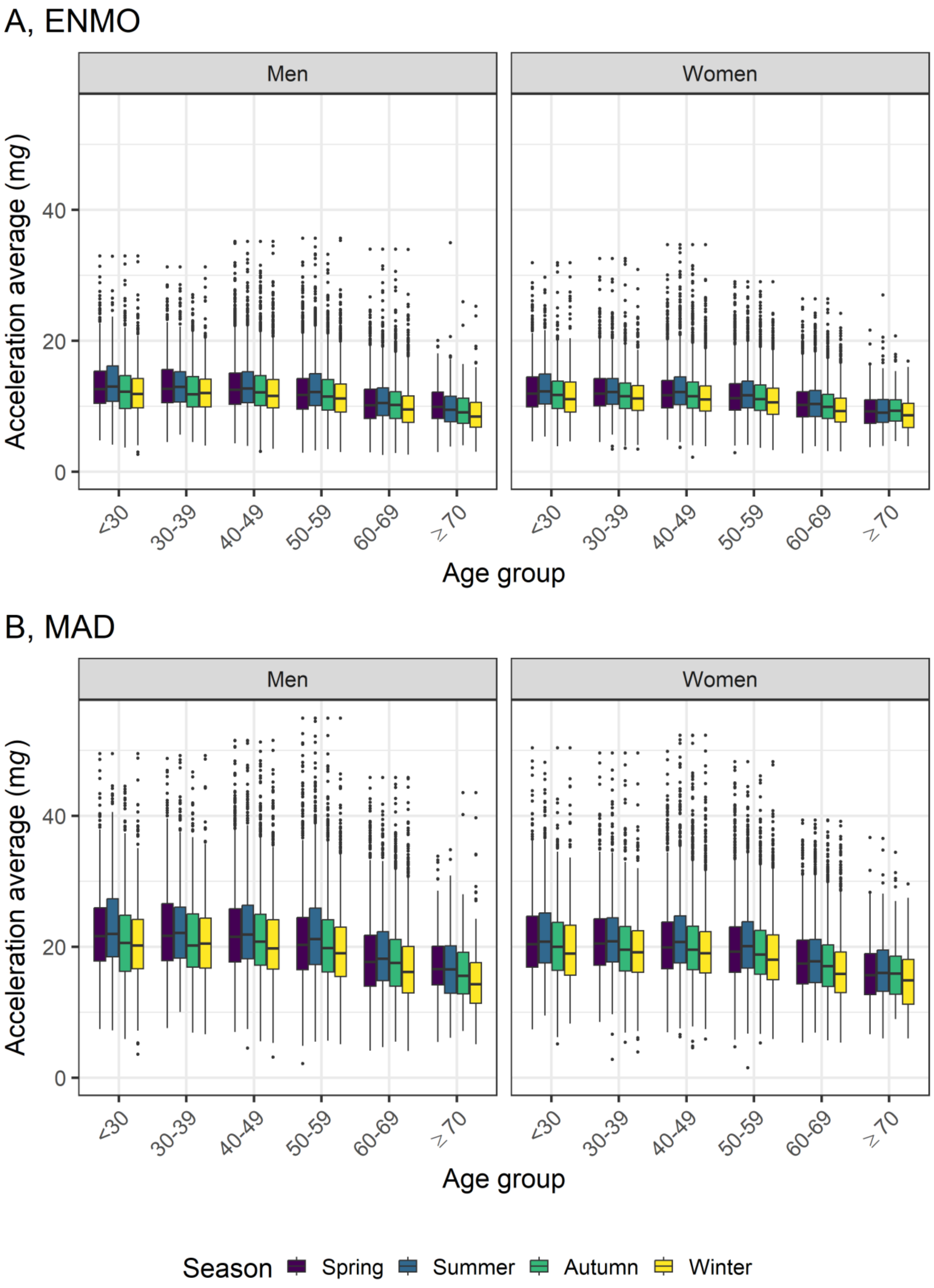

Figure S4 Seasonal variation in magnitude of acceleration A, ENMO and B, MAD by season, age, and sex

ENMO = Euclidean Norm Minus One with negative values set to zero; MAD = Mean Amplitude Deviation; m*g* = milli gravitational acceleration
n=63,236
ENMO and MAD values were winsorized at age- and sex-specific 99.9th percentile

Interpretation of box and whiskers plot: The box depicts the interquartile range (IQR, central 50% of the distribution) with the 25% quantile and the 75% quantile as lower and upper limits, respectively, as well as the median (50% quantile, middle line); the lower whisker shows the smallest observation that is greater than or equal to the 25% quantile - 1.5 * IQR; the upper whisker depicts the largest observation that is less than or equal to the 75% quantile + 1.5 * IQR; the dots indicate outliers beyond the whiskers.

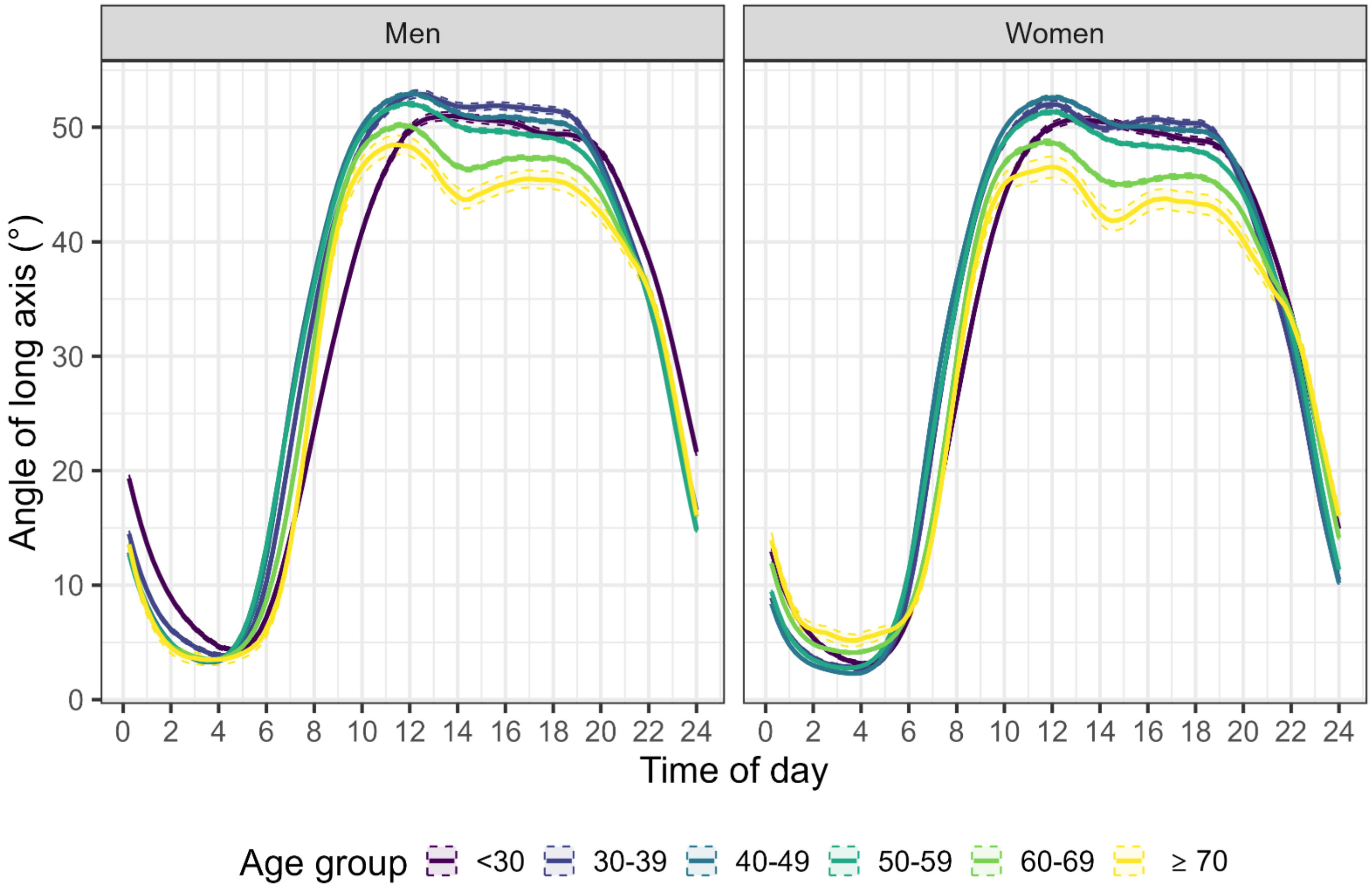

Figure S5 Accelerometer orientation: variation in angle of long axis across the day by age and sex

n=63,236

Shading bounds represent two standard errors.

The angle indicates the absolute angle relative to the horizontal plane of the accelerometer axis that aligns best with the participant's longitudinal body axis. Specifically, lower values indicate that the participant is lying horizontally and higher values indicate that the participant is sitting or standing upright.

The calculation of the angles is implemented in the function g.applymetrics (https://github.com/wadpac/GGIR/blob/master/R/g.applymetrics.R) of the R package GGIR, and the main formula was previously reported,[3] but does not detail the resampling and filtering steps prior to applying the formula as can be found in the code.

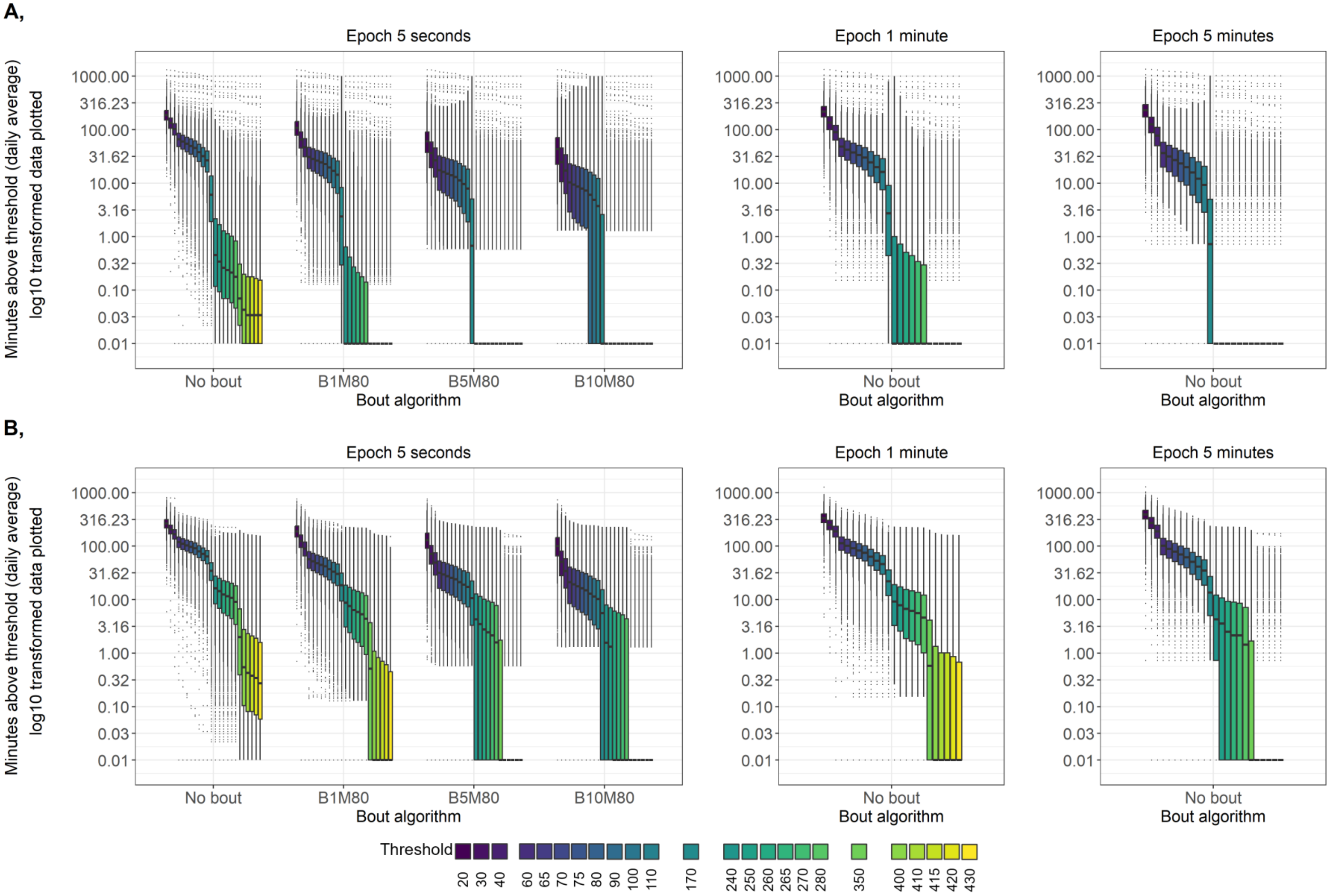

Figure S6 Comparison of time spent above various thresholds based on A, ENMO and B, MAD using different epoch lengths and bout algorithms

ENMO = Euclidean Norm Minus One with negative values set to zero; MAD = Mean Amplitude Deviation; B1M80, B5M80 and B10M80 see Box S1
n=63,236; Values were not winsorized
Interpretation of box and whiskers plot: The box depicts the interquartile range (IQR, central 50% of the distribution) with the 25% quantile and the 75% quantile as lower and upper limits, respectively, as well as the median (50% quantile, middle line); the lower whisker shows the smallest observation that is greater than or equal to the 25% quantile - 1.5 * IQR; the upper whisker depicts the largest observation that is less than or equal to the 75% quantile + 1.5 * IQR; the dots indicate outliers beyond the whiskers.

Box S1 Parameters and thresholds used to estimate time spent above threshold values

Parameters employed:

- Acceleration summary metrics:
  - ENMO
  - MAD
- Epoch length (Time interval over which the raw data are aggregated):
  - 5 seconds
  - 1 minute
  - 5 minutes
- Bout-algorithm:
  - No bout: counting each epoch above the threshold criterion
  - B1M80: bouts of at least 1 minute where at least 80% of epochs must meet the threshold criteria
  - B5M80: bouts of at least 5 minutes where at least 80% of epochs must meet the threshold criteria
  - B10M80: bouts of at least 10 minutes where at least 80% of epochs must meet the threshold criteria
- Thresholds: selection of published cut points and adjacent thresholds for sensitivity analyses, e.g.,
  - Hildebrand 2014/2016:[4,5] adults (21-61 years), ActiGraph, hip, ENMO: Light: 47.4 m*g*, Moderate: 69.1 m*g*, Vigorous: 258.7 m*g*
  - Sanders 2019:[6] older adults (60-68 years), ActiGraph, hip, ENMO: Light 6[a]/15[b] m*g,* Moderate 19[a]/69[b] m*g*
  - Migueles 2021:[7] older adults (≥70 years), ActiGraph, hip, ENMO: Light 7 m*g*, Moderate 14 m*g*
  - Vähä-Ypyä 2015:[8] adults (35 +/- 11 years), Hookie AM20, hip, MAD: Moderate: 91 m*g*, Vigorous: 414 m*g*

ENMO = Euclidean Norm Minus One with negative values set to zero; MAD = Mean Amplitude Deviation; m*g* = milli gravitational acceleration

[a] when applying the Youden index on ROC curves.

[b] when increasing Sensitivity over Specificity for light and vice versa for moderate physical activity on ROC curves.

Table S2 Checklists for transparent documentation of raw accelerometry quality control[9]

Checklist for data management and quality control		
1	Screen data for accelerometer non-wear periods	Yes
2	Screen data for implausible data points	Yes
3	Inspect data for acceleration sensor calibration error, e.g., relative to gravitational acceleration	Yes
4	Verify sensor attachment orientation if the algorithm depends on it	Yes
5	Keep a record of data cleaning at study level, e.g., individuals excluded	Yes
6	Keep a record of data cleaning at individual level, e.g., measurement periods included/excluded	Yes
7	Keep a record of the algorithm version(s) used	Yes
Checklist for reporting on data processing		
1	Report source and developer of employed algorithm(s)	Yes
2	Report parameters and coefficient values used for the algorithm(s)	Yes
3	Report on programming environment specifications	Yes
4	Provide a written description and motivation of the key steps in the algorithm	Yes
5	Provide, where possible, a reference to other publications using the same algorithm	Yes
6	Provide, where possible, literature references for studies supporting the appropriateness of the algorithm for application under the conditions for which it is used	Yes

References:

1. van Hees VT, Gorzelniak L, Dean Leon EC, Eder M, Pias M, Taherian S et al. Separating movement and gravity components in an acceleration signal and implications for the assessment of human daily physical activity. PLoS One. 2013;8(4):e61691. doi:10.1371/journal.pone.0061691.

2. Aittasalo M, Vähä-Ypyä H, Vasankari T, Husu P, Jussila AM, Sievänen H. Mean amplitude deviation calculated from raw acceleration data: a novel method for classifying the intensity of adolescents' physical activity irrespective of accelerometer brand. BMC sports science, medicine and rehabilitation. 2015;7:18. doi:10.1186/s13102-015-0010-0.

3. van Hees VT, Sabia S, Jones SE, Wood AR, Anderson KN, Kivimäki M et al. Estimating sleep parameters using an accelerometer without sleep diary. Scientific Reports. 2018;8:12975. Doi:10.1038/s41598-018-31266-z.

4. Hildebrand M, Hansen BH, van Hees VT, Ekelund U. Evaluation of raw acceleration sedentary thresholds in children and adults. Scandinavian journal of medicine & science in sports. 2016. doi:10.1111/sms.12795.

5. Hildebrand M, van Hees VT, Hansen BH, Ekelund U. Age group comparability of raw accelerometer output from wrist- and hip-worn monitors. Medicine and science in sports and exercise. 2014;46(9):1816-24. doi:10.1249/mss.0000000000000289.

6. Sanders GJ, Boddy LM, Sparks SA, Curry WB, Roe B, Kaehne A et al. Evaluation of wrist and hip sedentary behaviour and moderate-to-vigorous physical activity raw acceleration cutpoints in older adults. Journal of sports sciences. 2019;37(11):1270-9. doi:10.1080/02640414.2018.1555904.

7. Migueles JH, Cadenas-Sanchez C, Alcantara JMA, Leal-Martín J, Mañas A, Ara I et al. Calibration and Cross-Validation of Accelerometer Cut-Points to Classify Sedentary Time and Physical Activity from Hip and Non-Dominant and Dominant Wrists in Older Adults. Sensors (Basel). 2021;21(10). doi:10.3390/s21103326.

8. Vähä-Ypyä H, Vasankari T, Husu P, Mänttäri A, Vuorimaa T, Suni J et al. Validation of Cut-Points for Evaluating the Intensity of Physical Activity with Accelerometry-Based Mean Amplitude Deviation (MAD). PloS one. 2015;10(8):e0134813. doi:10.1371/journal.pone.0134813.

9. van Hees VT, Thaler-Kall K, Wolf KH, Brond JC, Bonomi A, Schulze M et al. Challenges and Opportunities for Harmonizing Research Methodology: Raw Accelerometry. Methods of information in medicine. 2016;55(6):525-32. doi:10.3414/me15-05-0013.
